# Monitoring HIV Drug Resistance Early Warning Indicators in Cameroon: A Study Following the Revised World Health Organization Recommendations

**DOI:** 10.1371/journal.pone.0129210

**Published:** 2015-06-17

**Authors:** Joseph Fokam, Jean-Bosco N. Elat, Serge C. Billong, Etienne Kembou, Armand S. Nkwescheu, Nicolas M. Obam, André Essiane, Judith N. Torimiro, Gatien K. Ekanmian, Alexis Ndjolo, Koulla S. Shiro, Anne C. Z-K. Bissek

**Affiliations:** 1 Chantal BIYA International Reference Centre for research on HIV/AIDS prevention and management (CIRCB), Yaoundé, Cameroon; 2 Faculty of Medicine and Biomedical Sciences (FMBS), University of Yaoundé I, Yaoundé, Cameroon; 3 University of Rome Tor Vergata, Department of Experimental Medicine and Surgery, Rome, Italy; 4 National HIV Drug Resistance Surveillance and Prevention Working Group, Ministry of Public Health, Yaoundé, Cameroon; 5 National AIDS Control Committee, Ministry of Public Health, Yaoundé, Cameroon; 6 World Health Organization (WHO), Afro, Country Office, Yaoundé, Cameroon; 7 Division of Operational Health Research, Ministry of Public Health, Yaoundé, Cameroon; 8 United Nations program on HIV/AIDS (UNAIDS), Yaoundé, Cameroon; 9 General Secretariat, Ministry of Public Health, Yaoundé, Cameroon; 10 Agence National de Recherche sur le SIDA et les hépatites virales (ANRS), Yaoundé, Cameroon; University of Athens, Medical School, GREECE

## Abstract

**Background:**

The majority (>95%) of new HIV infection occurs in resource-limited settings, and Cameroon is still experiencing a generalized epidemic with ~122,638 patients receiving antiretroviral therapy (ART). A detrimental outcome in scaling-up ART is the emergence HIV drug resistance (HIVDR), suggesting the need for pragmatic approaches in sustaining a successful ART performance.

**Methods:**

A survey was conducted in 15 ART sites of the Centre and Littoral regions of Cameroon in 2013 (10 urban versus 05 rural settings; 8 at tertiary/secondary versus 7 at primary healthcare levels), evaluating HIVDR-early warning indicators (EWIs) as-per the 2012 revised World Health Organization’s guidelines: EWI_1_ (*on-time pill pick-up*), EWI_2_ (*retention in care*), EWI_3_ (*no pharmacy stock-outs*), EWI_4_ (*dispensing practices*), EWI_5_ (*virological suppression*). Poor performance was interpreted as potential HIVDR.

**Results:**

Only 33.3% (4/12) of sites reached the desirable performance for *“on-time pill pick-up”* (57.1% urban versus 0% rural; p<0.0001) besides 25% (3/12) with fair performance. 69.2% (9/13) reached the desirable performance for *“retention in care”* (77.8% urban versus 50% rural; p=0.01) beside 7.7% (1/13) with fair performance. Only 14.4% (2/13) reached the desirable performance of *“no pharmacy stock-outs”* (11.1% urban versus 25% rural; p=0.02). All 15 sites reached the desirable performance of 0% *“dispensing mono- or dual-therapy”*. Data were unavailable to evaluate *“virological suppression”* due to limited access to viral load testing (min-max: <1%-15%). Potential HIVDR was higher in rural (57.9%) compared to urban (27.8%) settings, p=0.02; and at primary (57.9%) compared to secondary/tertiary (33.3%) healthcare levels, p=0.09.

**Conclusions:**

Delayed pill pick-up and pharmacy stock-outs are major factors favoring HIVDR emergence, with higher risks in rural settings and at primary healthcare. Retention in care appears acceptable in general while ART dispensing practices are standard. There is need to support patient-adherence to pharmacy appointments while reinforcing the national drug supply system.

## Introduction

By end 2012, ~9.7 million individuals were receiving antiretroviral therapy (ART) in low- and middle-income countries (LMIC), representing ~60% and 30% of global ART coverage in adult and pediatric populations, respectively [1]. Importantly, >95% of new human immunodeficiency virus (HIV) infection occurred in LMICs [2]. Among LMICs, sub-Saharan Africa (SSA) is the most affected by HIV/AIDS (67%-71% of the global epidemics from 2010–2013), in spite of the increasing access to ART (>62% coverage) in this region [2–3].

With the target of 15 million individuals on ART by 2015 [1], the wide use of low genetic-barrier drugs and the recently revised guidelines for earlier ART initiation (CD4<500 cells/μl), scale-up of ART would drive faster, suggesting upward risks of HIV drug resistance (HIVDR) emergence in SSA [4].

Based on recent evidences supporting optimal surveillance, care and prevention strategies, the 2012 updated HIVDR global strategy of the World Health Organization (WHO) now shelters five distinct components, among which: (a) the threshold of transmitted HIVDR in recently infected populations; (b) HIVDR in populations initiating ART; (c) acquired HIVDR in populations receiving ART; (d) initial HIVDR among children aged <18 months; (e) monitoring HIVDR early warning indicators (EWI) [3,4]. The last component, known to be a pragmatic and low-cost approach, is very efficient to support the performance of ART programs in LMICs [5–8].

As several SSA countries, Cameroon is still experiencing a generalized HIV epidemiology (4.3% prevalence in ~20 million inhabitants), with ~122,638 patients receiving ART [min-max: 117,998–122,856] (accounting for ~50% treatment eligible patients in 2013, up from 2% in 2003) [9–11]. In addition to this rapid scale-up of ART, poor adherence, lost to follow-up and pharmacy stock-outs have been addressed nationwide [7–8], as well as low-moderate levels of HIVDR in ART-naïve populations [12–14] and increasing rates with treatment-experience [15]. It is therefore necessary to monitor and evaluate country performance, following lessons and challenges in the ART program [7,14], and provide evidence for public health actions [3,16].

Although HIVDR is inevitable, surveillance strategies using EWIs have help limiting the spread of preventable HIVDR patterns in several LMICs [17–18]. Moreover, there are limited reference laboratory facilities and genotypic resistance testing is generally for population-based HIVDR surveillance [3–5,19]. In this context, EWI would efficiently optimize local HIV treatment performance [3,6].

Following the 2012 revision, five simplified EWIs are currently suitable to evaluate clinic and programmatic factors significantly associated with HIVDR [6], among which EWI_1_: *“* ***on-time pill pick-up*** *”* (previously referred to as “*on-time pill pick-up*” or as “*on-time clinic appointment keeping*”, or as “*pill count or standardized adherence measure*”); EWI_2_: “***retention in care at 12 months***” (previously referred to both as “*retention on appropriate first-line ART at 12 months*” and as “*lost to follow-up at 12 months*”); EWI_3_: “***pharmacy stock-outs***” (previously referred to as “*pharmacy stock-outs”*); EWI_4_: “***dispensing practices***” (previously referred to as “*prescribing practices*”), EWI_5_: “***virological suppression***” (previously referred same as “*virological suppression*”) [6,20].

Based on: (a) our national HIVDR working group experience in monitoring EWI [7,8], (b) our contribution to the Lusaka resolutions related to challenges in previous EWIs (Zambia, Lusaka, 2012 meeting), and (c) our commitment to the global resistance surveillance network (WHO visioconference, Switzerland, Geneva, August 2013), it was deemed relevant to evaluate the feasibility of the revised EWIs [6], through a pilot study, for strategic planning, monitoring and evaluation of the national ART program.

## Materials and Methods

### Study design

A retrospectively designed survey was conducted during the months of February and March 2014 to evaluate HIVDR-EWIs accounting for the year 2013 in 15-selected sentinel ART sites in Cameroon, within the reporting period of October 2012 to September 2013, including an additional three months (October—December 2013) to allow time to enter site and abstract data, as well as allowing sufficient timing for patients requiring further monitoring to complete a 12-month follow-up schedule.

### Sampling procedure of ART sites

For the first attempt in evaluating these revised EWIs, this pilot study was planned in sampled ART sites located in the Centre and Littoral regions of Cameroon, following the WHO *“* ***primary sampling strategy*** *”* for monitoring EWI [6]. These regions represent 20% of national regions, and are the most experienced on ART management (≥10 years) as compared to other regions. Of note, the Centre and littoral region have respectively the political and economic capital cities, and accounting for 33% of the national demography [9,10,21]. Selected as the most appropriate national settings, representativeness in these regions was ensured using a selective scoring system: (i) the clinic experience on ART (<3 years = 0, 3–5 years = 1 point, 5–7 years = 2 points, >7 years = 4 points); (ii) number of patients newly enrolled on ART per trimester (< 30 = 0, 30–40 = 1 point, 40–50 = 2 points, 50–60 = 3 points, 60–80 = 4 points, >80 = 5 points); (iii) levels of healthcare delivery (primary [i.e. basic level health facilities] = 2 points, secondary [i.e. intermediate level in the health facilities] = 4 points, tertiary [i.e. specialized health facilities] = 5 points); (iv) geographic location (rural or urban = 5 points); and (v) clinic affiliation (public, private or religious) [6]. ART clinics were ranked and those with the highest scores selected as sentinel sites (Table 1).

**Table 1:** List of Selected Sites and their Required Sample Size for the 2013 EWI Survey (Adopted from the WHO EWI HIVDR Guidelines, 2010).

Region	N°	Name of the selected sites	Geographic location of site (Urban or Rural)	Healthcare category (Primary, Secondary, or Tertiary)	Patients enrolled on ART in 2012	Sample size (WHO guidelines)
**Centre**	**1**	**Yaoundé Central Hospital**	**Urban**	**Secondary**	**1921**	**180**
	**2**	**National Social Welfare Hospital**	**Urban**	**Secondary**	**796**	**160**
	**3**	**Yaoundé Jamot Hospital**	**Urban**	**Secondary**	**938**	**175**
	**4**	**Yaounde General Hospital**	**Urban**	**Tertiary**	**421**	**140**
	**5**	**Yaoundé Pediatric and Gyneco-Obstetric Hospital**	**Urban**	**Tertiary**	**263**	**120**
	**6**	**University Health Centre—Yaoundé**	**Urban**	**Tertiary**	**634**	**155**
	**7**	**Cité Verte District Hospital**	**Urban**	**Primary**	**435**	**145**
	**8**	**Mbalmayo District Hospital**	**Rural**	**Primary**	**323**	**130**
	**9**	**Obala District Hospital**	**Rural**	**Primary**	**254**	**120**
**Littoral**	**10**	**Douala Laquintinie Hospital**	**Urban**	**Secondary**	**615**	**155**
	**11**	**Douala General Hospital**	**Urban**	**Tertiary**	**377**	**135**
	**12**	**Nylon District Hospital**	**Urban**	**Primary**	**958**	**175**
	**13**	**Saint jean Malte Hospital of Njombe**	**Rural**	**Primary**	**260**	**120**
	**14**	**Nkongsamba District Hospital**	**Rural**	**Primary**	**381**	**135**
	**15**	**Edéa District Hospital**	**Rural**	**Primary**	**292**	**120**
**Total**						**2165**

EWI, early warning indicator; WHO, World Health Organization; HIVDR, HIV drug resistance; ‘‘Urban” referred to city/township settings; ‘‘Rural” referred to peripheral/village settings; ‘‘Primary” referred to first-level health facilities; ‘‘Secondary” referred to medium/intermediate level health facilities; ‘‘Tertiary” referred to high/reference level health facilities.

### EWI definition, Data Abstraction and Required Targets

At the level of each site, data clerks performed collection of data from ART registers, referred to as “data abstraction”, following standard criteria (Table 2). Abstraction tools (in English and French versions) are provided as supporting information (S1 Tables).

**Table 2:** Definition of EWIs and their respective performance targets.

EWI and title	Definition	Numerator	Denominator	Target
**EWI**_**1**_: **On-time pill pick-up.**	Proportion of patients (adult or children) that pick-up ART no more than two days late at the first pick-up after the baseline pick-up.	Number of patients picking-up their ART “on time” at the first drug pick-up after baseline pick-up date.	Number of patients who picked-up ARV drugs on or after the designated EWI sample start date.	Desirable performance (green): >90%; fair performance (amber): 80–90%; poor performance (red): <80%.
**EWI**_**2**_: **Retention in care.**	Percentage of adults and children known to be alive and on ART 12 months after initiation.	Number of adults (or children) who are still alive and on ART 12 months after initiating treatment.	Total number of adults or children (excluding transfers out) who initiated ART and were expected to achieve 12-month outcomes within the reporting period.	Desirable performance (green): >85%; fair performance (amber): 75–85%; poor performance (red): <75%.
**EWI**_**3**_: **Pharmacy stock-outs.**	Percentage of months in a designated year in which there were no ARV drug stock-outs (both for adult and pediatric patients).	Number of months in the designated year in which there were no stock-out days of any ARV drug routinely used at the site.	12 months of the reporting period.	Desirable performance (green): 100%; poor performance (red): <100%.
**EWI**_**4**_: **Pharmacy dispensing practice.**	Percentage of patients (adults or children) being dispensed a mono- or dual-ART.	Number of patients who pick up from the pharmacy, a regimen consisting of one or two ARVs.	Number of patients picking up ART on or after the designated EWI sample start date	Desirable performance (green) defined as 0% patients picking-up a mono- or dual-ART; poor performance (red) defined as >0% patients picking-up a mono- or dual-ART.
**EWI**_**5**_: **Virological suppression.**	Percentage of patients (adult or children) receiving ART at the site after the first 12 months of ART whose viral load is <1000 copies/ml.	Number of patients receiving ART at the site after the first 12 months of ART whose viral load is <1000 copies/ml.	Number of patients at the site who by national policy should have had a viral load performed 12 months after ART initiation.	Desirable performance (green): >85%; fair performance (amber): 70–85%; poor performance (red): <70%.

EWI, early warning indicator

EWI_4_ is cross sectional in nature and is intended to assess pharmacy dispensing practices for populations on ART after any period of time on ART.

### Sampling timeframe for EWI data abstraction

Sampling timeline was based on a 12-month “reporting period” (i.e. October 2012—September 2013) required to assess “***retention in care***”, “***pharmacy stock-out***” and “***virological suppression***”; while “***on-time pill pick-up***” and “***dispensing practices***” required a cross-sectional data abstraction following a consecutive enrollment.

### Quality Assurance and Data Analysis

Prior to data collection, site staff was trained on EWIs and methodology for data abstraction. Data were abstracted from ART registers, pharmacy stock records and/or patient medical records (for assessing VL) using standardized EWI abstraction sheets (S1 Tables).

To minimize bias in the entire dataset, data abstracted onsite were reviewed and validated (using 10% data) by supervisors. Data were then centralized at the national level, entered on a predefined Excel spreadsheet (*routine data quality assessment [RDQA]* tool) following the 2012 WHO-EWI guidelines, for final validation.

Threshold of each EWI was analyzed and performance was interpreted according to defined target (Table 2): desirable (green), fair (amber) or poor (red) performance. Clinics presenting a poor performance for any EWI were interpreted as sites with potential HIVDR. Fisher exact test was used for categorical data analysis, and p-values <0.05 were considered statistically significant.

### Ethical Considerations

The present survey was implemented by the Cameroonian Ministry of Public Health, through the Division of health operational research (i.e. the national regulatory authority in health research), and with support from the WHO. The Ministry of Public Health provided administrative authorization (N°D84-20/MP/MINSANTE/SESP/SG/DROS/CRS/CEA1), following the application N°194-13/NHA/MINSANTE/SG/DROS/CRC/CEA2/AP*;* waiving ethical clearance and informed consent as-per the retrospective design and the routine landscape of this public health surveillance activity using de-identified archived data and conducted for regular performance evaluation of the national ART program.

## Results

### Description of ART clinics selected as sentinel sites

All selected ART sites participated in the survey, among which 9 in the Centre and 6 in the Littoral regions of Cameroon. According to geographical locations, there were 10 urban versus 5 rural sites. According to levels of healthcare, there were 7 sites at primary, 4 sites at secondary and 4 sites at tertiary levels. Up to 14 sites belonged to the public and only one from the religious sector. First-line ART regimens were prescribed in all sites while second-line regimens were offered exclusively at secondary or tertiary healthcare levels (Table 1).

A therapeutic committee (a weekly consultative body for ART initiation made of clinicians, pharmacists/clerks, medical biologists/technicians, nurses, counselors) was present in all sites but poorly functioning. ART registers were available (not always standard across sites) but poorly handled some sites. By end 2013, 8,868 patients were receiving ART in all the entire sites, with only 26.7% (4/15) of sites having an electronic software (i.e. *ESOPE* approved by the Ministry of Public Health, *SUPATARV* or Excel spreadsheets) for routine monitoring of patients on ART.

### Levels of HIVDR EWIs

#### Overall EWI-based Performance of ART sites

A total of 2165 ARV-treated adult patients were effectively enrolled, as-per statistical requirement (Table 1). Overall, desirable performances were reported in 33.3% of ART sites for EWI_1_ *(* ***on-time pill pick-up***), 69.2% for EWI_2_ *(* ***retention in care 12 months after initiation*** *)*, 14.4% for EWI_3_ *(* ***no pharmacy stock-outs*** *)* and 100% for EWI_4_ (***no dispensing of mono- or bi-therapy***); data were not available for EWI_5_ (***virological suppression***). Thus, potential HIVDR was driven by delayed pill pick-up and frequent pharmacy stock-outs (Table 3).

**Table 3:** Overall Performance of EWIs.

EWI	EWI_1_			EWI_2_			EWI_3_		EWI_4_		EWI_5_
**Performance**	Desirable	Fair	^❖^Poor	Desirable	Fair	^❖^Poor	Desirable	^❖^Poor	Desirable	^❖^Poor	NA
**Sites (%)**	33,3% (4/12)	25% (3/12)	41,6% (5/12)	69,2% (9/13)	7,8% (1/13)	23% (3/13)	14,4% (2/14)	85,6% (12/14)	100% (15/15)	0% (0/15)	NA

NA, Not available;

^❖^Poor performance interpreted as “Potential HIVDR”

#### Performance for each EWI

**Fig 1:**
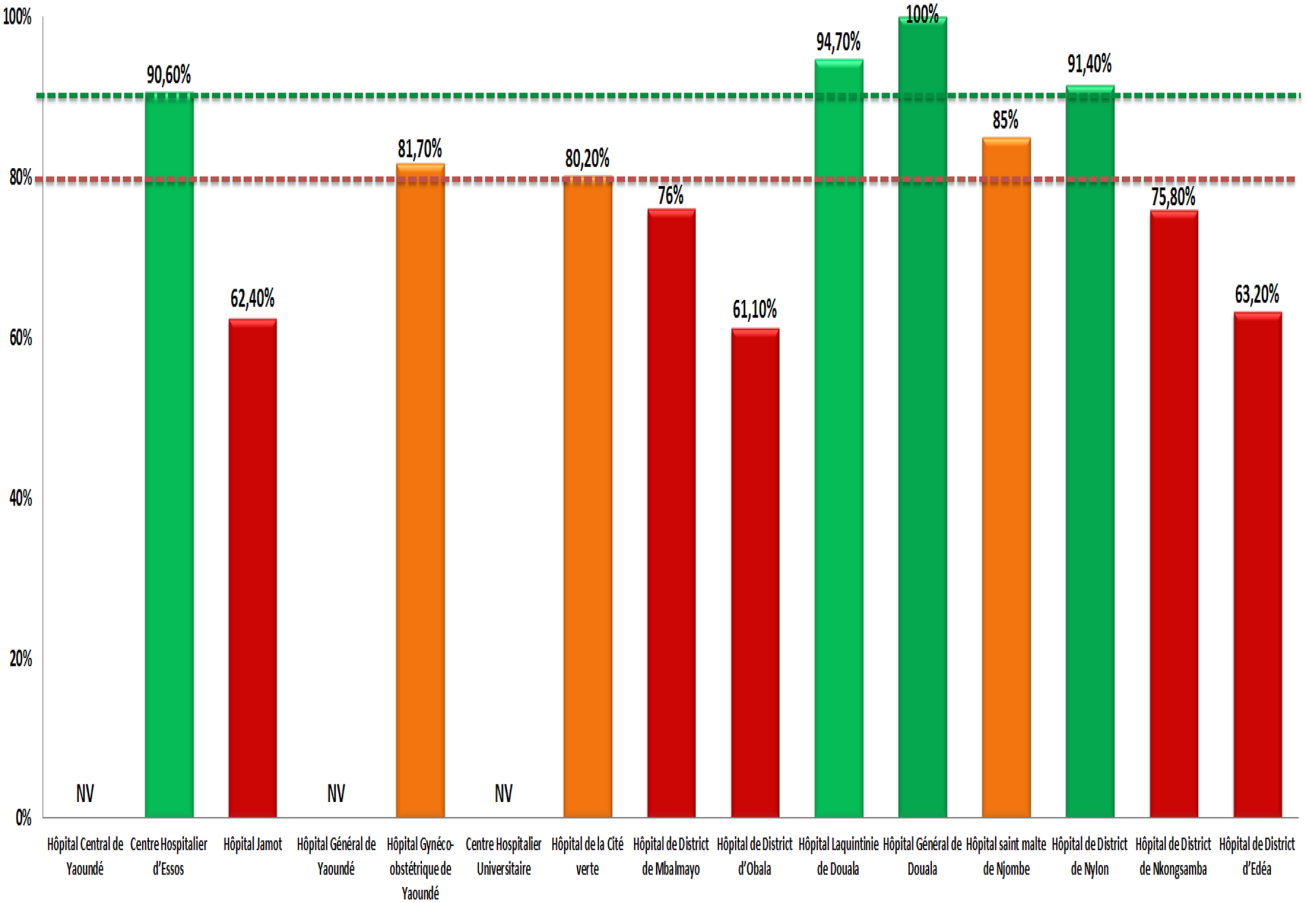
Site Performance for “On-time Pill Pick-up” in 2013. NV, Not Validated; green, desirable performance; amber, fair performance; red, poor performance. Horizontal lines indicate the lower limit thresholds for “desirable” (in “green” color) and for “fair” (in “amber” color) performance.

For EWI_1_ *(* ***on-time pill pick-up)***, data from three sites were invalid (due to inconsistent reporting in the ART register). Only 33.3% (4/12) sites with desirable performance of >90%, 25% (3/12) with fair performance (80–90%), against 41.7% (5/12) (<80%) with poor performance (Fig 1). For entire dataset, see supporting information (S2 and S3 tables).

**Fig 2:**
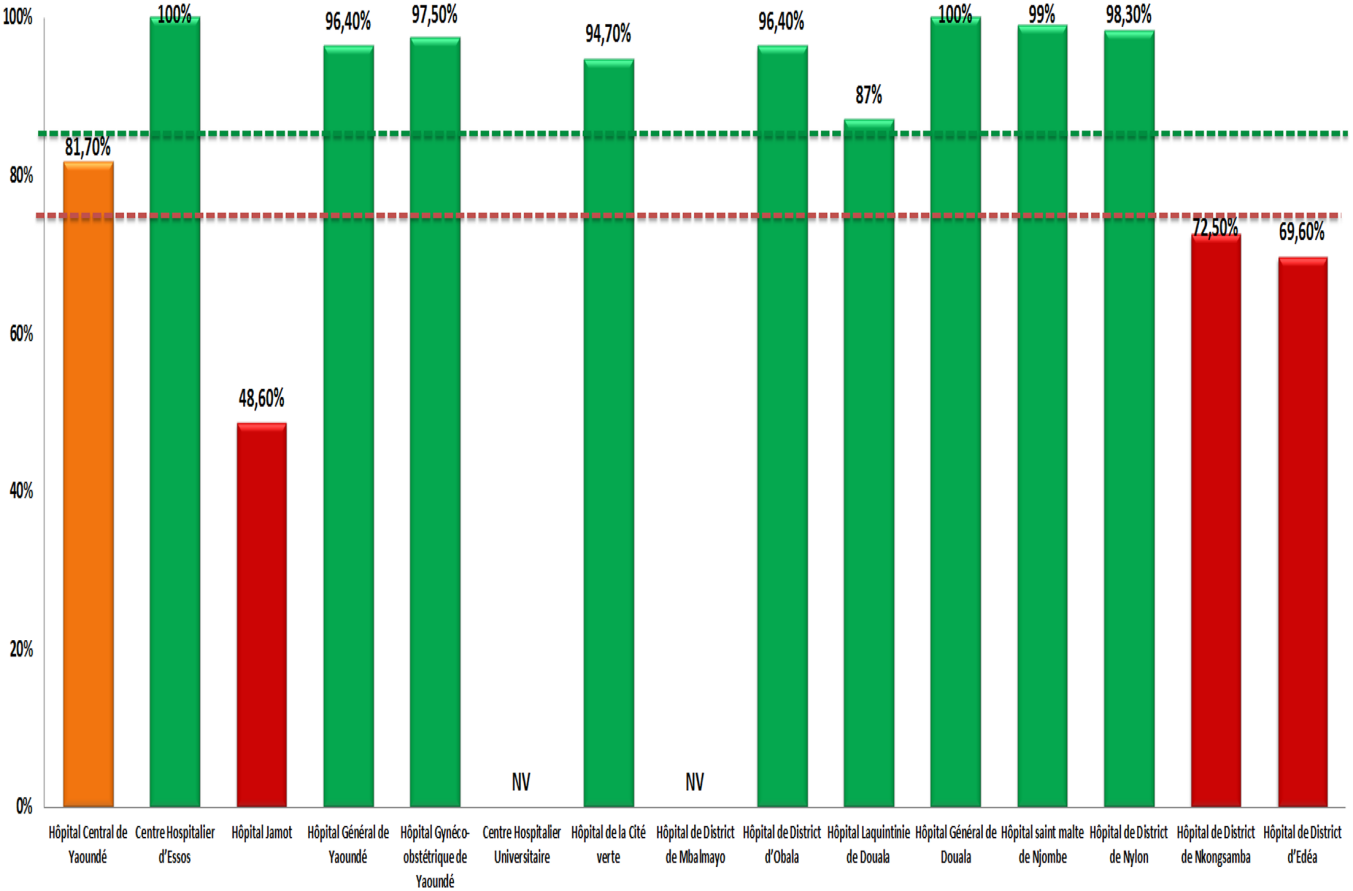
Site Performance for “Retention in Care 12 months after ART Initiation” in 2013. NV, Not Validated; green, desirable performance; amber, fair performance; red, poor performance. Horizontal lines indicate the lower limit thresholds for “desirable” (in “green” color) and for “fair” (in “amber” color) performance.

For EWI_2_ ***(retention in care 12 months after initiation)***, data from two sites were invalid. Up to 69.2% (09/13) sites with desirable performance of 85%, 7.7% (01/13) with fair performance of 75–85%, against 23.1% (03/13) with poor performance (min-max: 48.6%-72.5%), as shown in Fig 2.

**Fig 3:**
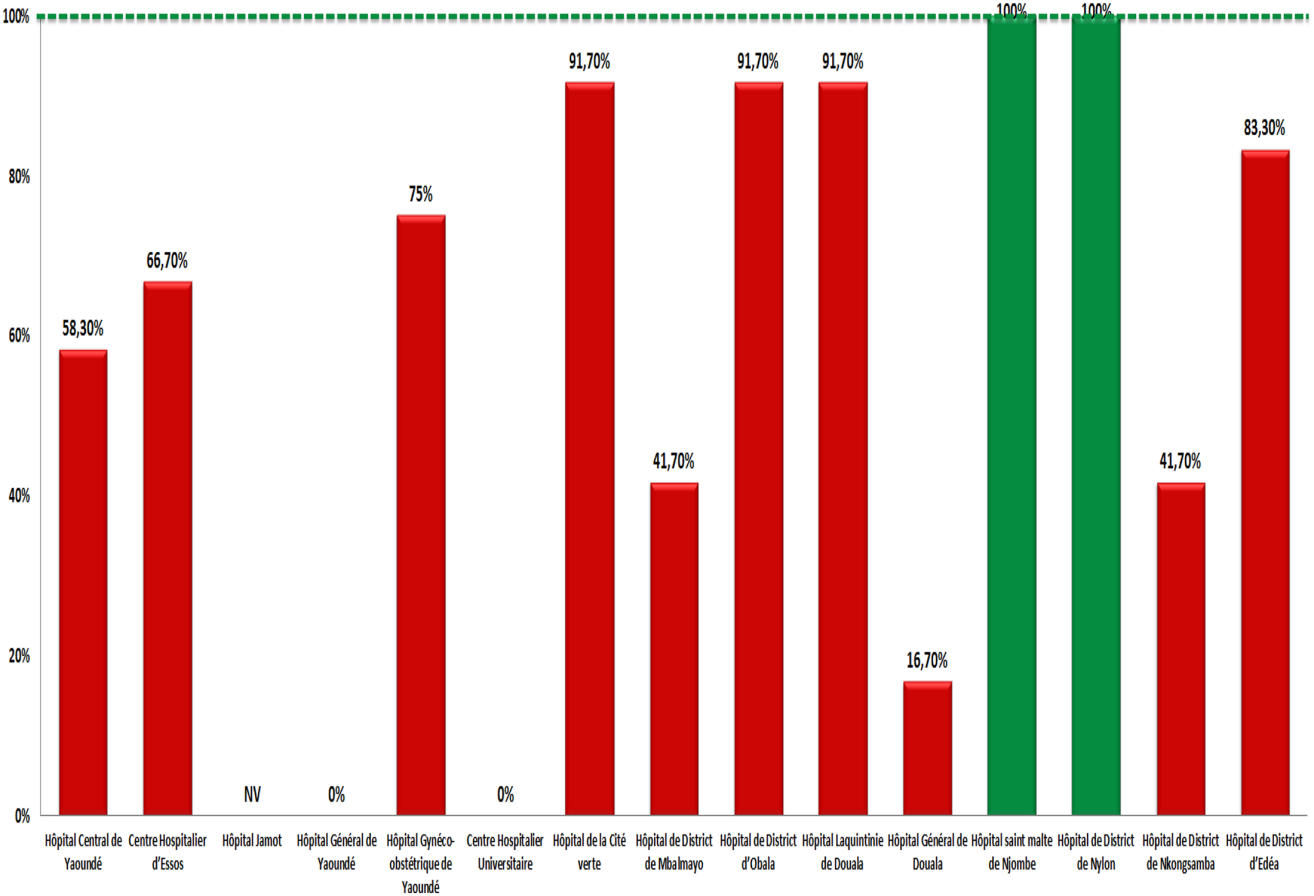
Site Performance for “No Pharmacy ARV Stock-outs” in 2013. NV, Not Validated; green, desirable performance; red, poor performance. The horizontal line in “green” color indicates the lower limit threshold for a “desirable” performance.

For EWI_3_, ***(no pharmacy drug stock-outs during 12 months)*** data from one site was invalid. Only 14.4% (2/14) sites with desirable performance, against 85.6% (12/14) on poor perfromance (min-max: 16.7%-91.7%), as shown in Fig 3.

For EWI_4_ ***(dispensing practices of mono- or bi-therapy)***, all 15 (100%) sites reached the desirable performance of 0% mono or bi-therapy, indicating dispensing and prescribing practices according to national guidelines.

For EWI_5_ (***virological suppression at 12 months ART)***, none of the sites reached the required sample size (i.e. ≥90% eligible patients with VL coverage at 12 months of ART) for evaluation. Only 20% (03/15) of sites had a laboratory facility for HIV VL measurement, operating intermittently. VL testing, ranging from <1% (mainly in rural sites) to 15% (in some urban sites), was mainly to confirm ART failure among patients with immunological/clinical failure. Thus, EWI_5_ evaluation was not feasible.

#### Comparison of EWI Performance between Urban and Rural Sites

With the exception of EWI_4_ (*dispensing practices*) reaching desirable target performance in all sites, higher rates of poor performance were reported in rural (57.9%) as compared urban (27.8%) settings; p = 0.02. Specifically, for EWI_1_ *(on-time pill pick-up)*, desirable performance was significantly higher in urban (57.1%) as compared to rural (0.0%) settings, p< 0,0001; similar for EWI_2_ *(retention in care after 12 months of initiation)* with 57.1% in urban and 0.0% in rural settings, p = 0.01. In contrast, EWI_3_ *(no drug stock-outs)* was significantly higher (p = 0.02) in rural settings (Table 4). Overall, potential HIVDR was higher in rural settings.

**Table 4:** Comparison of EWIs Performance between Urban and Rural Sites.

EWI	Target performance	Urban Sites (10)	Rural Sites (05)	P-value
**EWI**_**1**_	Desirable (>90%),	57.1% (4/7)	0% (0/5)	< 0.0001
	Fair (80–90%)	28.6% (2/7)	20% (1/5)	0.05
	^❖^Poor (<80%)	14.3% (1/7)	80% (4/5)	0.0001
**EWI**_**2**_	Desirable (>85%)	77.8% (7/9)	50% (2/4)	0.01
	Fair (75–85%)	11.1% (1/9)	0% (0/4)	0.4
	^❖^Poor (<75%)	11.1% (1/9)	50% (2/4)	0.0001
**EWI**_**3**_	Desirable (100%)	11.1% (1/9)	25% (1/5)	0.02
	^❖^Poor (<100%)	88.9% (8/9)	75% (4/5)	0.22
**EWI**_**4**_	Desirable (0%)	100% (10/10)	100% (5/5)	0.9
	^❖^Poor (>0%)	0% (0/10)	0% (0/0)	1
**EWI**_**5**_	NA	NA	NA	NA

EWI, early warning indicator; NA, Not Available; ‘‘Urban” referred to city/township settings; ‘‘Rural” referred to peripheral/village settings;

^❖^Poor performance interpreted as “Potential HIVDR”.

#### Comparison of EWI Performance according to Levels of Healthcare Delivery

With the exception of EWI_4_ (dispensing practices) reaching desirable target performance in all sites, higher rates of poor performance were reported at primary (57.9%) as compared to secondary/tertiary (33.3%) healthcare levels; p = 0.09. Specifically, for EWI_1_ *(on-time pill pick-up)*, desirable performance was significantly higher at secondary/tertiary healthcare (60.0%) as compared to primary (14.3%) healthcare levels (p = 0.001), with a similar trend observed for EWI_2_ *(retention in care 12 months after ART initiation)*; p = 0.005. In contrast, desirable performance for EWI_3_ (*no drug stock-outs*) was significantly higher at the level of primary healthcare (28.6%) as compared to secondary/tertiary (0%) levels, p = 0.03 (Table 5). Overall, potential HIVDR was higher at the level of primary healthcare.

**Table 5:** Comparison of EWI Performance between the Primary versus Secondary/Tertiary Levels.

EWI	Target performance	Secondary/Tertiary Levels (08)	Primary Level (07)	P-value
**EWI**_**1**_	Desirable (>90%)	60% (3/5)	14.3% (1/7)	0.001
	Fair (80–90%)	20% (1/5)	28.6% (2/7)	0.7
	^❖^Poor (<80%)	20% (1/5)	57.1% (4/7)	0.00002
**EWI**_**2**_	Desirable (>85%)	71.4% (5/7)	66.7% (4/6)	0,8
	Fair (75–85%)	14.3% (1/7)	0% (0/6)	< 0.0001
	^❖^Poor (<75%)	14.3% (1/7)	33.3% (2/6)	0.005
**EWI**_**3**_	Desirable (100%)	0% (0/7)	28.6% (2/7)	0.03
	^❖^Poor (<100%)	100% (7/7)	71.4% (5/7)	0.0289
**EWI**_**4**_	Desirable (0%)	100% (8/8)	100% (7/7)	0.99
	^❖^Poor (>0%)	0% (0/0)	0% (0/0)	1
**EWI**_**5**_	NA	NA	NA	NA

EWI, early warning indicator; NA, Not available; ‘‘Primary” referred to first-level health facilities; ‘‘Secondary” referred to medium/intermediate level health facilities; ‘‘Tertiary” referred to high/reference level health facilities;

^❖^Poor performance interpreted as “Potential HIVDR”.

## Discussion

With the goal to optimize patient care and to prevent HIVDR in Cameroon, we effectively monitored EWIs as-per the latest WHO recommendations, and identified clinics and programmatic factors with sub-optimal performance. Of note, majority of ART clinics experienced delay in drug pick-up, indicating the need to support adherence to pharmacy appointments. This performance was significantly poor in rural settings and at primary healthcare deliveries, possibly favored by the poor transportation systems [7,8]. As HIVDR is likely to emerge more in these settings, a priority plan for adherence support should be implemented for patients attending rural or primary healthcare clinics [21], as recently addressed in Namibia for this EWI (42% in adults and 49% in pediatrics) [22].

Retention in care was satisfactory in about 2/3 of surveyed sites, suggesting that many ART clinics in these Cameroonian regions may have the capacity to sustain patients on ART; a performance higher in urban settings, likely due to easier access to health facilities and better transportation system. However, further studies would provide distinct variability between settings at the national level, as well as delineating poor retention due to lost-to-follow-up, unreported deaths, ART interruption, etc. [7,8]. Re-defining the role played by community health agents might improve long-term performance [23]. Variable performances were reported between adults (45%) and children (57%) in Namibia, [22], attaining up to 80.2% in other SSA-settings [23].

Drug stock-outs were frequent, especially in urban and secondary/tertiary healthcare facilities, partly attributed to the growing burnout syndrome (heavy workload) in the cities and the eventual ineffectiveness of the drug management system [7,8]. Of note, only sites receiving supports from partners (i.e. “*Doctors without Borders*” and the only religious clinic) did not experience any stock out [7,8]. Though several SSA-countries encountered similar challenges [18, 24], the Namibian experience (90% and 76% months without adult and pediatric stock-outs, respectively) suggests improving inventory management, storage space, avoiding short-dated ARVs and inappropriate estimates, with procurement/supply under constant supervision [4,22,25].

No case of mono- or dual-therapy was identified, suggesting low risk of HIVDR, mainly due to simplified/standard regimens (fixed-dose combinations) and the role of therapeutic committees [4,10,19], suggesting sharing of experience to support performance in Namibia (96% in adults and 89% in children) [7,8, 22].

The poor coverage of VL testing was due to limited laboratory facility, lack of manpower, and high costs for VL testing (USD $40–85 per test in other settings) [26]. VL testing was mainly performed when suspecting treatment failure (CD4 decline or poor clinical outcome), mainly at patient’s cost, (range: $33 – $55) and performed at reference laboratories. Current efforts in cost reduction by partners ($20 per test) should supplement by the government with “*point-of-care”* VL assays ($10–20 per test), especially at the periphery [27–28]. While requesting for ART registers to be standardized to capture VL data, evaluating EWI_5_ at the moment is not yet feasible.

The major limitation of our study is the number study sites (15 sentinel sites with 2165 patients on ART), which weakens the scope of representativeness. Nonetheless, such pilot study, prior to larger surveys, gives a clue on the feasibility of revised EWIs. Of note, we have confirmed the persistent drug stock-outs, good dispensing practices, and poor virological monitoring in Cameroon. Thus, further studies would now focus on delineating retention in care [7–8], besides evaluating the relevance in setting-up a national task force on antimicrobial resistance [27–30].

## Conclusions

The revised set of EWIs is feasible at 80% (4/5) in ART-programs facing similar challenges like Cameroon, thus recommending scale-up of VL monitoring. Frequent drug stock-outs and delayed pill pick-up are major factors favoring risks of preventable HIVDR emergence, especially at the periphery. While implementing corrective measures, further studies are needed to better outline patient retention in care.

## Supporting Information

S1 Tables EWI-Abstraction-Tools French-English. (DOC)

S2 Tables EWI Centre region sites. (ZIP)

S3 Tables EWI Littoral region sites. (ZIP)
