# Supplementary material for: Monitoring HIV Drug Resistance Early Warning Indicators in Cameroon: A Study Following the Revised World Health Organization Recommendations
Source: PLoS One. 2015 Jun 17;10(6):e0129210. doi: 10.1371/journal.pone.0129210 (PMC4471113; doi:10.1371/journal.pone.0129210)
Supplement: S1 Tables — (DOC) [file pone.0129210.s001.doc]

| INDICATEURS d’ALERTE PRECOCE DE LA PHARMACORESISTANCE DU VIH - Abstraction de données OUTIL 1 | | | | | |
| --- | --- | --- | --- | --- | --- |
|  |  | | | |  |
| Indicateur 1 : Délais de retrait des Médicaments (IAP1) - Pourcentage de patients ayant retiré tous les ARV prescrits dans les délais | | | | |  |
| **1. Pays:** | 2. Formation sanitaire ou site: | | | |  |
| **3.** Date d'abstraction (extraction) de données**:** | 4. Nombre total de feuilles (pour les données extraites sur papier seulement): |  | 4. Nombre de patients non classés |  | |
| **5. D**ate du début de l'échantillon de l’IAP (JJ -MM- AA) : | |  |  |  | |
| **6.** Nombre total de patients éligibles: |  | 8. Taille minimale requise de l'échantillon: (Si vous utilisez la feuille d'abstraction imprimé, se référer au document guide des IAP pour la taille minimale requise de l'échantillon) | |  | |
| **7.** Extracteur des données (nom / prénom / contact téléphonique): |  | | |  |  |

| **No.** | Code ID du Patient (à noter que l’IAP 1 comprend tous les patients sous TARV dans le site, y compris ceux débutant le TARV, continuant le TARV, et les transferts-in sous TARV) | **Date de retrait de base** (la date du premier retrait du médicament ARV après "la date de début de l’échantillon de l’IAP’’, cf.n ° 5 ci-dessus) | Protocol ARV retiré au "retrait de base" (ARV tel qu’enregistré dans le registre de la pharmacie) | Nombre de jours de TARV reçu à la " Date du retrait base " - si nécessaire, calculer à partir du nombre de comprimés et la masse (mg), et comprimés/jour en utilisant la calculatrice de conversion; ou les informations enregistrées dans la colonne précédente. | Nombre de jours avec TARV restant - si enregistré et applicable. | Retrait 1 (date du premier retrait d’ARV après " la date du retrait de base ") – noter la date de retrait du nouveau protocole d’ARV si changement de TARV après la date du retrait de base | Date de transfert après " " la date du retrait de base "  (si applicable) | Date de décès après " " la date du retrait de base "  (si applicable) | Date d’arrêt du TARV sans reprise après " " la date du retrait de base "  (si applicable) |
| --- | --- | --- | --- | --- | --- | --- | --- | --- | --- |
|  |  |  |  |  |  |  |  |  |  |
|  |  |  |  |  |  |  |  |  |  |
|  |  |  |  |  |  |  |  |  |  |
|  |  |  |  |  |  |  |  |  |  |
|  |  |  |  |  |  |  |  |  |  |
|  |  |  |  |  |  |  |  |  |  |
|  |  |  |  |  |  |  |  |  |  |

|  |  |  |  | |  |  |  |  | |  | |  | | | | | |  |
| --- | --- | --- | --- | --- | --- | --- | --- | --- | --- | --- | --- | --- | --- | --- | --- | --- | --- | --- |
|  | **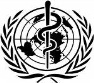**  INDICATEURS d’ALERTE PRECOCE DE LA PHARMACO RESISTANCE DU VIH - abstraction de données OUTIL 2  (adulte et pédiatrique)   |  | | --- | | | |  | | | | **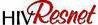** | | | | |  | |  |  | | |
|  |  | | |  | | | |  | | | | |  | |  |  | | |
|  | Indicateur: rétention sous TARV (IAP 2) - Pourcentage d'adultes et d'enfants connus vivant et sous traitement 12 mois après l’initiation du TARV | | | | | | | | | | | | | | |  | | |
|  |  | | |
|  | **1. Pays:** | | | **2.** Formation sanitaire ou site: | | | | | | | | | | | |  | | |
|  | **3.** Date d'abstraction (extraction) de données**:** | | |  | | | **4.** Nombre total de feuilles (pour les données extraites sur papier seulement): | | | | | |  | |  |  | | |
|  |  | | | **5. D**ate du début de l'échantillon de l’IAP (JJ -MM- AA) : | | |  | | | | | |  | |  |  | | |
|  | **6.** Nombre total de patients éligibles: | | |  | | |  | | | | | |  | |  |  | | |
|  | **7.** Extracteur des données (nom / prénom / contact téléphonique): | | | | | |  | | | | | |  | |  |  | | |
|  |  | | |  | | |  | | | | | |  | |  |  | | |
|  | **UNGASS #24 / PEPFAR #T1.3.D / Global Fund Impact #HIV-I3 Site Result** | | | | | | REMARQUE: Les patients adultes et pédiatriques sont combinés pour cet indicateur. Entrée le numérateur et le dénominateur de UNGASS / PEPFAR / Fonds mondial dans les cellules appropriées. Si l’extraction de l’IAP n’est pas réalisé durant l'année de reportage d'UNGASS, alors entrer le résultat des données les plus récentes en matière de rétention sur le site. | | | | | | | | |  | | |
|  | **Numérateur** | | |  | | |  | | |
|  | **Dénominateur** | | |  | | |  | | |
|  | Résultat du Site | | |  | | |  | | |
|  |  | | |  | | |  | | | | | |  | |  |  | | |
|  |  | | |  | | |  | | | | | |  | |  |  | | |
|  |  | | |  | | |  | | | | | |  | |  |  | | |
|  |  | | |  | | |  | | | | | |  | |  |  | | |
|  |  | | |  | | |  | | | | | |  | |  |  | | |
|  |  | | |  | | |  | | | | | |  | |  |  | | |
|  | 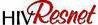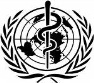   | INDICATEURS d’ALERTE PRECOCE DE LA PHARMACO RESISTANCE DU VIH - abstraction de données OUTIL 3 (Adulte) | | --- | | | |  | | |  |  |  | |  |  |  |  |  |  |  | |

|  |  |  |  |  |  |  |  |  |  |  |  |  |
| --- | --- | --- | --- | --- | --- | --- | --- | --- | --- | --- | --- | --- |
|  | Indicateur: La continuité de l'approvisionnement en médicaments (IAP 3) - Pourcentage de mois au cours d’une année donnée sans ruptures de stock d’ARV | | | | | | | | | |  |  |
|  |  |  |
|  | **1. Pays:** |  | **2.** Facilité ? (Installation ?? lieu ?? établissement ??)): | | | |  |  |  |  |  |  |
|  | **3.** Date d'abstraction (extraction) de données**:** |  | | **4.** Nombre total de feuilles (extraction sur papier): | | |  | | | |  |  |
|  | **5. Période de remplissage (1 an) :** |  | |  | | |  |  |  |  |  |  |
|  | **6.** Extracteur des données (nom / prénom / contact téléphonique): | | |  |  |  |  |  |  |  |  |  |
|  | *Pour chaque médicament antirétroviral utilisé en routine sur le site, entrez un "X" dans le menu déroulant pour chaque mois au cours duquel il y avait au moins une rupture de stock d'un médicament ARV utilisés en routine sur ce site. Lister chaque ARV utilisé en routine sur le site, même si aucune rupture de stock d'un médicament n’a eu lieu au cours de la période désignée.* | | |  |  |  |  |  |  |  |  |  |

| **No.** | Médicaments antirétroviraux | **Jan** | **Feb** | **Mar** | **Apr** | **May** | **Jun** | **Jul** | **Aug** | **Sep** | **Oct** | **Nov** | **Dec** |
| --- | --- | --- | --- | --- | --- | --- | --- | --- | --- | --- | --- | --- | --- |
|  |  |  |  |  |  |  |  |  |  |  |  |  |  |
|  |  |  |  |  |  |  |  |  |  |  |  |  |  |
|  |  |  |  |  |  |  |  |  |  |  |  |  |  |
|  |  |  |  |  |  |  |  |  |  |  |  |  |  |
|  |  |  |  |  |  |  |  |  |  |  |  |  |  |
|  |  |  |  |  |  |  |  |  |  |  |  |  |  |
|  |  |  |  |  |  |  |  |  |  |  |  |  |  |

| 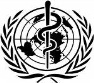  INDICATEURS d’ALERTE PRECOCE DE LA PHARMACO RESISTANCE DU VIH - abstraction de données OUTIL **4 (Adulte)**   |  | | --- | | |  | | --- | | |  | | **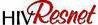** |
| --- | --- | --- | --- | --- | --- | --- | --- |
|  | Indicateur: Pratiques de dispensation (IAP4) - Pourcentage de patients adultes ayant retiré des ARV en mono ou bithérapie | | | | |
|  | **1. Pays:** | **2.** Formation sanitaire ou site : | | | |
|  | **3.** Date d'abstraction (extraction) de données**:** |  | | **4.** Nombre total de feuilles (pour les données extraites sur papier seulement): | |
|  |  | **5. D**ate du début de l'échantillon de l’IAP (JJ -MM- AA) | |  | |
|  | **6.** Nombre total de patients éligibles: |  | |  | |
|  |  | **7.** Extracteur des données (nom / prénom / contact téléphonique): | |  | |
| **No.** | Code ID du patient (à noter que l’IAP 4 concerne tous les patients sous TARV dans le site, y compris ceux initiant le TARV, continuant le TARV, et les transferts in sous TARV) | Protocole ARV retiré à la date du mois de base | | Protocole thérapeutique Mono ou Dual ? | |
|  |  |  | |  | |
|  |  |  | |  | |
|  |  |  | |  | |
|  |  |  | |  | |
|  |  |  | |  | |
|  |  |  | |  | |
|  |  |  | |  | |
|  |  |  | |  | |
|  |  |  | |  | |
|  |  |  | |  | |
|  |  |  | |  | |

| 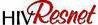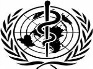 **HIV DRUG RESISTANCE EARLY WARNING INDICATORS - DATA ABSTRACTION TOOL 1 (Adult)** |  |  |  |  |  |
| --- | --- | --- | --- | --- | --- |
|  |  | | | |  |
| **Indicator: On-time Pill Pick-up (EWI 1) - Percentage of ART patients picking up all prescribed ARV drugs on time** | | | | |  |
| **1. Country:** | **2. Facility:** | | | |  |
| **3. Data abstraction date(s):** | **4. Total number of sheets** (for paper-based data abstraction only)**:** |  | **Number of patients not classified** |  | |
| **5. EWI sample start date(YY-MM-DD):** | |  |  |  | |
| **6. Total number of eligible patients:** |  | **8. Required minimum sample size: (**If using printed abstraction sheet, then refer to EWI Guidance document for required minimum sample size.) | |  | |
| **7. Data abstractor (name / last name / contact phone):** |  | | |  |  |

| **No.** | **Patient ID** (note that EWI 1 includes all patients on ART at the facility, including those initiating ART, continuing ART and transferring in on ART) | **Baseline pick-up** (the date of the first ARV drug pick-up after the "EWI sample start date", #5 above) | **ART regimen picked up at "baseline pick-up" (ARV drugs as recorded on pharmacy record)** | | **Number of days of ART picked up at "baseline pick-up" - if needed, calculate from pill number and strength (mg) and pills/day using Conversion Calculator; or record in previous column.** | **Number of days ART remnant - if recorded and applicable.** | **Pick-up 1** (date of first ARV drug pick-up after "baseline pick-up") - enter date of pick-up of new regimen if it was changed after the baseline pick-up date | **Date of transfer out after "baseline pick-up"** - if applicable | | **Date of death after "baseline pick-up"** - if applicable | | **Date of ART stop without restart after "baseline pick-up"** - if applicable | | | | | |
| --- | --- | --- | --- | --- | --- | --- | --- | --- | --- | --- | --- | --- | --- | --- | --- | --- | --- |
|  |  |  |  | |  |  |  |  | |  | |  | | | | | |
|  |  |  |  | |  |  |  |  | |  | |  | | | | | |
|  |  |  |  | |  |  |  |  | |  | |  | | | | | |
|  |  |  |  | |  |  |  |  | |  | |  | | | | | |
|  |  |  |  | |  |  |  |  | |  | |  | | | | | |
|  |  |  |  | |  |  |  |  | |  | |  | | | | | |
|  |  |  |  | |  |  |  |  | |  | |  | | | | | |
|  |  |  |  | |  |  |  |  | |  | |  | | | | | |
|  |  |  |  | |  |  |  |  | |  | |  | | | | | |
|  | |  | | --- | | | |  | | | |  | | | | |  | |  |  | |
| **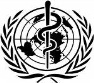** | **HIV DRUG RESISTANCE EARLY WARNING INDICATORS - DATA ABSTRACTION TOOL 2 (Adult & Paediatric)** | | |  | | | |  | | | | | **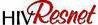** | |  |  | |
|  | **Indicator: Retention in Care (EWI 2) - Percentage of adults and children known to be alive and on treatment 12 months after initiation of ART** | | | | | | | | | | | | | | |  | |
|  |  | |
|  | **1. Country:** | | | **2. Facility:** | | | | | | | | | | | |  | |
|  | **3. Data abstraction date(s):** | | |  | | | **4. Total number of sheets** (for paper-based data abstraction only)**:** | | | | | |  | |  |  | |
|  |  | | | **5. EWI sample start date (YY-MM-DD):** | | |  | | | | | |  | |  |  | |
|  | **6. Total number of patients:** | | |  | | |  | | | | | |  | |  |  | |
|  | **7. Data abstractor (name / last name / contact phone):** | | | | | |  | | | | | |  | |  |  | |
|  |  | | |  | | | **NOTE: Adult & paediatric patients combined for this indicator. Input UNGASS/ PEPFAR/ Global Fund numerator and denominator in appropriate cells. If EWI abstraction not done during UNGASS reporting year, then enter result for most recent site retention results.** | | | | | |  | |  |  | |
|  | **UNGASS #24 / PEPFAR #T1.3.D / Global Fund Impact #HIV-I3 Site Result** | | | | | |  | | | | | | | | |  | |
|  | **Numerator** | | |  | | |  | |
|  | **Denominator** | | |  | | |  | |
|  | **Site Result** | | |  | | |  | |
|  |  | | |  | | |  | | | | | |  | |  |  | |
|  |  | | |  | | |  | | | | | |  | |  |  | |
|  |  | | |  | | |  | | | | | |  | |  |  | |
|  |  | | |  | | |  | | | | | |  | |  |  | |
|  |  | | |  | | |  | | | | | |  | |  |  | |
|  |  | | |  | | |  | | | | | |  | |  |  | |
|  | |  | | --- | | | |  | | |  |  |  | |  |  |  |  |  |  |  |
|  |  | | |  | | |  |  |  | |  |  |  |  |  |  |  |
|  |  | | |  | | |  |  |  | |  |  |  |  |  |  |  |
| **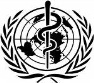** | **HIV DRUG RESISTANCE EARLY WARNING INDICATORS - DATA ABSTRACTION TOOL 3 (Adult)** | | |  | | |  |  |  | |  |  | 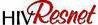 |  |  |  |  |
|  | **Indicator: Drug supply continuity (EWI 3) - Percentage of months in a designated year in which there were NO ARV drug stock-outs** | | | | | | | | | | | | | | |  |  |
|  |  |  |
|  | **1. Country:** | | |  | | | **2. Facility:** | | | | |  |  |  |  |  |  |
|  | **3. Data abstraction date:** | | |  | | | | **4. Total number of sheets (**paper-based abstraction)**:** | | | |  | | | |  |  |
|  | **5. Reporting Period:** | | |  | | | |  | | | |  |  |  |  |  |  |
|  | **6. Data abstractor (name / last name / contact phone):** | | | | | | |  |  | |  |  |  |  |  |  |  |
|  | ***For each ARV drug routinely used at the site, enter an "X" from the drop-down menu for each month in which there was at least one stock-out of ANY ARV drug routinely used at this site. List each ARV drug routinely used at the site, even if no stockouts of a drug occurred during the designated time period.*** | | | | | | |  |  | |  |  |  |  |  |  |  |

| **No.** | **Antiretroviral drug** | **Jan** | **Feb** | **Mar** | **Apr** | **May** | **Jun** | **Jul** | **Aug** | **Sep** | **Oct** | **Nov** | **Dec** |
| --- | --- | --- | --- | --- | --- | --- | --- | --- | --- | --- | --- | --- | --- |
|  |  |  |  |  |  |  |  |  |  |  |  |  |  |
|  |  |  |  |  |  |  |  |  |  |  |  |  |  |
|  |  |  |  |  |  |  |  |  |  |  |  |  |  |
|  |  |  |  |  |  |  |  |  |  |  |  |  |  |
|  |  |  |  |  |  |  |  |  |  |  |  |  |  |
|  |  |  |  |  |  |  |  |  |  |  |  |  |  |
|  |  |  |  |  |  |  |  |  |  |  |  |  |  |

| 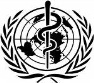   |  | | --- | | | **HIV DRUG RESISTANCE EARLY WARNING INDICATORS - DATA ABSTRACTION TOOL 4 (Adult)** | | --- | | |  | | **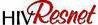** |
| --- | --- | --- | --- | --- | --- | --- | --- |
|  | **Indicator: Dispensing Practices (EWI 4) - Percentage of adult patients picking up mono or dual ARV therapy** | | | | |
|  | **1. Country:** | **2. Facility:** | | | |
|  | **3. Data abstraction date(s):** |  | | **4. Total number of sheets** (for paper-based data abstraction only)**:** | |
|  |  | **5. EWI sample start date(YY-MM-DD):** | |  | |
|  | **6. Total number of eligible patients:** |  | |  | |
|  |  | **7. Data abstractor (name / last name / contact phone):** | |  | |
| **No.** | **Patient ID** (note that EWI 4 includes all patients on ART at the facility, including those initiating ART, continuing ART and transferring in on ART) | **ART regimen picked-up at baseline pick-up** | | **Mono or Dual Therapy Regimen?** | |
|  |  |  | |  | |
|  |  |  | |  | |
|  |  |  | |  | |
|  |  |  | |  | |
|  |  |  | |  | |
|  |  |  | |  | |
|  |  |  | |  | |
|  |  |  | |  | |
|  |  |  | |  | |
|  |  |  | |  | |
|  |  |  | |  | |
